# Role for cFMS in maintaining alternative macrophage polarization in SIV infection: implications for HIV neuropathogenesis

**DOI:** 10.1186/s12974-015-0272-1

**Published:** 2015-03-25

**Authors:** Lindsey Gerngross, Gabrielle Lehmicke, Aghilas Belkadi, Tracy Fischer

**Affiliations:** Department of Neuroscience, Center for Neurovirology, Temple University School of Medicine, MERB, Room 748, Philadelphia, PA 19140 USA

**Keywords:** M-CSF, IL-34, cFMS, M2, Macrophage, Alternative activation, CD163 HIV, SIV, Encephalitis

## Abstract

**Background:**

Macrophage-colony stimulating factor (M-CSF) has been implicated in HIV neuropathogenesis through its ability to modulate activation of macrophages (MΦs) and microglia, as well as enhance the susceptibility of these cells to infection and promote virus production. We have recently reported that MΦs accumulating perivascularly and within nodular lesions in archival brain tissue of simian immunodeficiency virus (SIV)-infected rhesus macaques with encephalitis (SIVE) express M-CSF. In contrast, IL-34, which shares the same receptor, cFMS, was observed more often in parenchymal cells.

**Methods:**

Frontal white and grey matter from non-infected and SIV-infected rhesus macaques with and without SIVE were examined by single- and double-label immunohistochemistry for M-CSF, IL-34, and CD163 expression. Primary rhesus macaque and human peripheral blood mononuclear cells were cultured with and without 2.5 ng/ml M-CSF or IL-34 alone and with 470 nM or 4.7 μM of GW2580, a receptor tyrosine kinase inhibitor with high specificity for cFMS. After 24 h, cells were analyzed by flow cytometry to examine the effect of these cytokines on promoting an M2 monocyte/MΦ phenotype.

**Results:**

Here, we demonstrate that in SIVE brain, accumulating M-CSF^+^ MΦs are also CD163^+^, while IL-34 does not appear to co-localize significantly with CD163 in the parenchyma. We further demonstrate that M-CSF and IL-34 are expressed by neurons in normal brain but are altered in SIV and SIVE. Through *in vitro* studies, we show that M-CSF and IL-34 upregulate CD163, a marker for type 2 activation of MΦs (M2), by primary monocytes, which is attenuated by the addition of GW2580.

**Conclusions:**

Together, these data suggest that both cFMS ligands may promote and/or prolong M2 activation of MΦs and microglia in brains of SIV-infected animals with encephalitis. As such, cFMS signaling may be an attractive target for eliminating long-lived MΦ reservoirs of HIV infection in brain, as well as other tissues.

## Background

Microglia and macrophages (MΦs) found within compartments associated with the central nervous system (CNS), such as perivascular MΦs, are essential to fighting off foreign pathogens, as well as maintaining CNS homeostasis. Injury to the CNS results in rapid activation of microglia and CNS-associated MΦs, which upregulate numerous cell-surface and soluble factors that trigger or amplify innate and acquired immune responses. The type, degree, and length of the microglial/MΦ response to injury can determine whether they are neuroprotective or contribute to neurodegeneration (for review, see Amor *et al*. [1]).

In addition to their pro-inflammatory functions and role in coordinating an adaptive immune response, tissue MΦs, including microglia, are involved in resolving inflammation and tissue remodeling and repair. To perform these varied functions, MΦ activation is considerably plastic and heterogeneous, broadly classified into two primary subclasses of classical (M1) and alternative (M2) activation, based on distinct functional and phenotypic characteristics (for review, see Liu *et al*. and Wang *et al*. [2,3]). Classically activated, pro-inflammatory M1 MΦs secrete interleukin (IL)-12 and support T helper (T_H_)1 cell responses [4]. In contrast, alternatively activated, anti-inflammatory M2 MΦs secrete IL-10, suppress T_H_1 function, and stimulate T_H_2 cell production [5,6]. A balance between classical and alternative activation is necessary for appropriate immune function and in a healthy immune response, MΦs are believed to rapidly shift from one activation state to another in response to external stimuli [7].

Although M2 MΦs are often considered ‘favorable’ due to their role in resolving inflammation, inducing tolerance, and promoting wound healing [6,8,9], an increase in the frequency of M2-polarized MΦs is believed to have injurious results associated with their transcriptional output in many chronic inflammatory conditions, including rheumatoid arthritis, atherosclerosis, and obesity [10-13]. In addition, extensive or prolonged M2 polarization may contribute to disease pathogenesis by weakening host immunity. For example, soluble factors produced by tumor-associated MΦs, which are reported to express markers consistent with M2-polarization [14], contribute to the impaired immune surveillance seen within the glioma microenvironment [15]. In neuroinflammatory disease, alternatively activated MΦs may be a continued source of reactive oxygen species (ROS) in the brain, which are necessary for conversion to M2 activation [16].

Many individuals with human immunodeficiency virus (HIV) infection develop varying degrees of cognitive impairment, collectively termed HIV-associated neurocognitive disorders (HAND). Although the pathogenesis of HAND is not completely understood, activated microglia and infiltrating MΦs into the CNS are believed to play a prominent role in its development and/or progression (for review, see Hong and Banks [17]). Recently, we reported mild-to-moderate accumulation of CD163^+^ perivascular MΦs and microglia in autopsy brain from anti-retroviral treated-HIV-infected persons without encephalitis, although to a much lesser degree than that seen in HIV encephalitis (HIVE), a histopathological correlate of the most severe form of HAND, HIV-associated dementia (HIV-D) [18]. This observation, as well as other indications of neuroinflammation in all HIV^+^ cases studied, suggests that chronic neuroinflammation, even in the absence of productive infection in the brain, is a common feature of HIV and likely contributes to all degrees of HAND.

Expression of CD163 has been used to phenotypically identify a subclass of M2 MΦs [19-22] (for review, see Moestrup and Møller [23]), suggesting that the observed CD163^+^ MΦs and microglia in brain are M2 activated/polarized. The inciting factor(s) of MΦ polarization in the context of HIV infection, however, is/are unknown. A leading candidate molecule in M2 polarization of MΦs in HIV infection is macrophage-colony stimulating factor (M-CSF). M-CSF has been shown in cerebrospinal fluid (CSF) of HIV-infected patients [24,25] and is believed to support virus production and disease progression (for review, see Haine *et al*. [26]). We recently identified the MΦs accumulating perivascularly and within nodular lesions as the principal source of M-CSF in simian immunodeficiency virus (SIV)-infected rhesus macaques, a relevant animal model for HIV-associated neuropathogenesis [27], with encephalitis (SIVE). In this same study, we found expression of IL-34, which shares the same receptor, cFMS, but appeared to have a greater association with cells in the parenchyma, rather than those accumulating perivascularly and within nodular lesions [27].

To further characterize these cells and explore the potential relationship of M-CSF and/or IL-34 with M2 activation, we performed immunohistochemical analyses of brain tissue from non-infected and SIV-infected rhesus macaques, with and without encephalitis (SIVE), to investigate the association between CD163 expression with M-CSF and IL-34. Here, we report that the CD163^+^ MΦs that accumulate perivascularly and within nodular lesions and serve as the primary reservoir of productive SIV and HIV infection in the CNS [28,29], also appear to be the primary source of M-CSF in SIVE. In contrast, CD163 expression in the parenchyma does not co-localize considerably with M-CSF, but does with IL-34. In addition, neurons also express M-CSF and IL-34, which is altered in SIV infection, suggesting a potential mechanism by which neurons communicate with neighboring microglia. Finally, our *in vitro* studies of rhesus macaque and human peripheral blood mononuclear cells (PBMC) demonstrate that cFMS signaling via either of its ligands may contribute to sustained M2 activation in brain in SIV infection and SIVE.

## Methods

### Immunohistochemistry

Sections from archival frontal lobe brain tissue (4 μm) of eight SIVmac251 infected and two non-infected rhesus macaques were kindly provided by Dr. Jay Rappaport. Sections include both frontal white matter (FWM) and frontal grey matter (FGM). Four of the eight SIV-infected animals had SIVE, characterized by the presence of perivascular cuffs, nodular lesions, and multinucleated giant cells, which are histological hallmarks of both HIV and SIV encephalitis [30-33]. Immunohistochemistry was performed as described by us previously [28,34,35]. Briefly, deparaffinized and rehydrated 4-μm brain tissue sections underwent high heat non-enzymatic antigen retrieval, followed by blocking with 20% normal goat (Lampire Biological Laboratories, Pipersville, USA) or horse (Fisher Scientific, Waltham, USA) serum and overnight incubation with primary mouse monoclonal CD163 (1:100; Vector Laboratories, Burlingame, USA), M-CSF (1:12.5; Novus Biologicals, Littleton, USA) or rabbit polyclonal IL-34 (1:200; Abcam, New Territories, Hong Kong) antibodies. The spleen from non-infected rhesus macaques was used as a positive control. Negative controls consisted of isotype antibodies used in place of the primary and tissues incubated in buffer without primary antibody. Antigen-specific staining was detected with goat-α-rabbit or horse-α-mouse biotinylated antibodies (Vector Laboratories), followed by Vectastain ABC Alkaline Phosphatase and Vector Red Alkaline Phosphatase Substrate Kit (Vector Laboratories), according to the manufacturer’s instructions. Tyramide signal amplification (Perkin Elmer, Waltham, USA) was used for CD163 and IL-34 detection, according to the manufacturer’s instructions. Following a light counterstain with hematoxylin, sections were dehydrated in xylenes, coverslipped with Permount, and analyzed under light microscopy. Immunohistochemistry for each antigen of interest was performed in a single run for all animals/groupings.

### Immunohistochemistry quantification

Quantification of single-label CD163, M-CSF, and IL-34 expression in brain among the three test animal groupings was completed using a bioquantification software system (Bioquant Image Analysis Program). A total of 12 ×20, microscopic fields of 0.31 mm^2^ each were assessed per brain section using a microscope (Nikon, Tokyo, Japan) with a motorized x, y stage and a digital camera (Q-Imaging Retiga, Surrey, Canada) that were linked to a computer with the software program. An unbiased quantification approach was used, with a random start, and then systematic sampling of 12 adjacent (non-overlapping) sites within the brain region of interest using the motorized stage option. Analysis conditions were retained across animals/groupings for each antigen of interest by white-balancing the camera prior to data acquisition and maintaining the same light intensity of the microscope for each slide. The Videocount Area Array and color thresholding options of the Bioquant software were utilized for these measurements, as previously defined in detail [36]. Briefly, videocount (VC) area is defined as the number of pixels in a field that meet a user-defined color threshold of staining. The user-defined color threshold of immunostaining for each antigen (that is, positivity of each antigen) was defined by setting the red-green-blue values to detect the range of positive signal values for a particular immunostained antigen, while excluding color ranges of other antigens and background noise. The threshold values for each antigen were stored in the computer program and maintained for each immunohistochemical analysis across all animals and groupings. The inclusion of positive cells and exclusion of potential background noise was verified by viewing the image of positive area projected onto the computer screen. Inappropriate inclusions/exclusions were corrected manually. The percent positivity for each field and antigen was determined by dividing the number of positive pixels (those that matched the defined color threshold) by the total number of pixels within the VC area, which were multiplied by 100. Data are expressed as the percentage of antigen positivity per 0.31 mm^2^ field. The assessment was carried out in a blinded fashion.

### Isolation of PBMC and cell culture

Whole blood from two non-infected, healthy rhesus macaques was acquired from Bioqual, Rockville, MD, USA, in accordance with Bioqual Institutional Animal Care and Use Committee protocol #14-3027-56. Buffy coat from a de-identified human volunteer was acquired from Biological Specialty Corporation in Colmar, PA, USA. The Biological Specialty Corporation is an FDA-licensed facility (US license #856) and abides by all applicable federal and state regulations concerning the collection and distribution of whole blood and blood products. PBMC were isolated by density gradient from heparinized blood or buffy coat using ACCUSPIN Tubes (Sigma, St. Louis, USA) containing Histopaque-1077 (Sigma). Viable PBMC were plated at 5 × 10^6^ cells/ml in Costar 24-well cell culture plates (Corning, Tewksbury, USA) and maintained in RPMI 1640 with L-glutamate (ThermoScientific, Waltham, USA) supplemented with penicillin/streptomycin (Cellgro, Manassas, USA) and 2% FBS. PBMC were maintained at 37°C and 5% CO_2_ for 24 h in media with and without 2.5 ng/ml recombinant human (rh)M-CSF (R&D, Minneapolis, USA) or rhIL-34 (R&D) and with and without 4.7 μM or 470nM GW2580 (Calbiochem, Billerica, USA). Brefeldin A (eBioscience, San Diego, USA) was added to each well 6 h prior to harvest. PBMC were harvested and washed with FACS Wash (FW; Hanks’ Balanced Salt Solution (ThermoScientific), 3% normal horse serum (NHS) (ThermoScientific), 0.02% sodium azide (Fisher)) and prepared for flow cytometric analysis.

### Flow cytometric analyses

**Figure 1:**
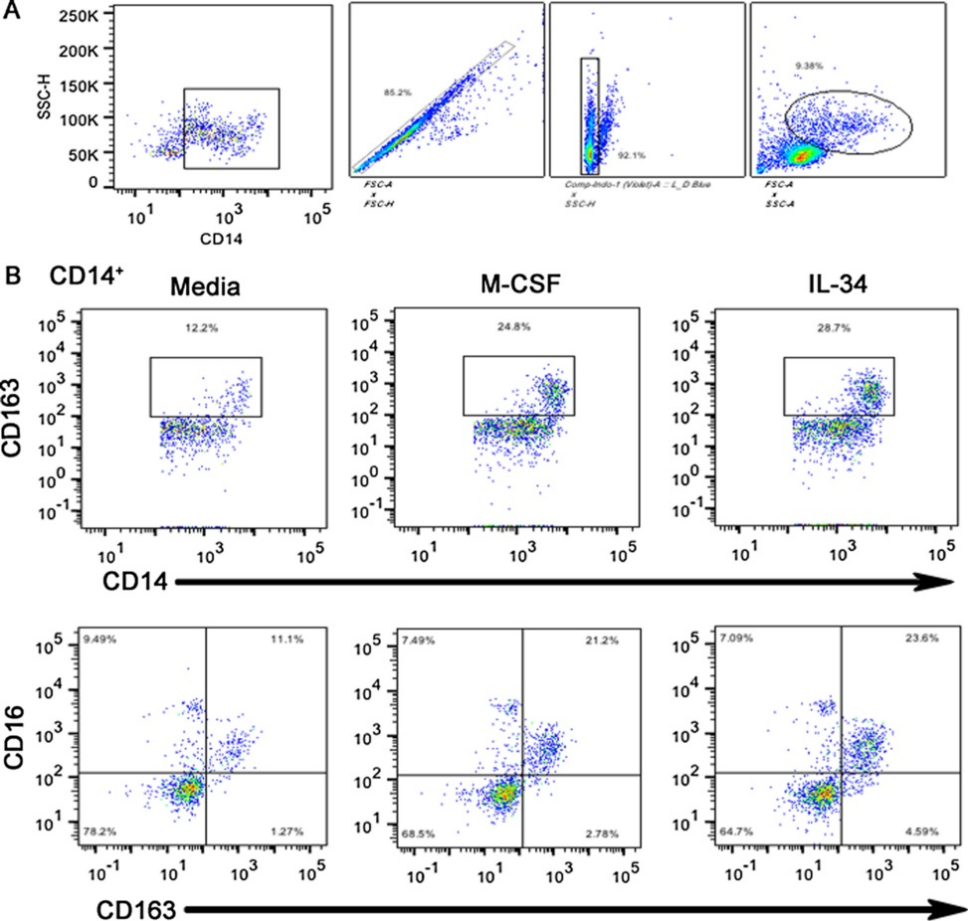
**CD14** ^**+**^ **back gating and dot plots of monocyte populations. (A)** Backgating of CD14^+^ population shows singlet isolation, identification of viable population, and forward and side scatter properties of parent population. CD14^+^ monocytes are the parent population for all monocyte populations/subsets described in this manuscript. **(B)** Representative dot plots demonstrate gating strategies used in identifying specific monocyte subsets under the different treatment conditions.

Flow cytometric analyses were performed on cultured human and rhesus macaque PBMC, using fluorochrome-conjugated antibodies, CD14-PCP-Cy5.5 (M5E2, BD, Franklin Lakes, USA), CD163-PE (Mac2-158; Trillium Diagnostics, Maine, USA), CD16-Pacific Blue (3G8, BD), and IL-10-APC (JES3-19 F1, BD) as described previously [37]. The antibody-fluorochrome panel was designed according to optimized detection and required information. Isotype controls were utilized in the construction of the panel ensure the specificity of each antibody in the panel. Fluorescence minus one (FMO) tests and non-stained PBMC were used for gating. For each test, 30,000 events were collected on a 4-laser 13-color LSRII Analyzer (BD) and analyzed using FlowJo version 10.0.7r2 (TreeStar, Ashland). Following identification of single events and live/dead discrimination using a Live/Dead Fixable Blue Dead Cell Stain Kit (Invitrogen, Grand Island, USA), monocytes were defined within a broad gate, based on forward scatter (FSC) and side scatter (SSC) properties. Identification of monocytes was further refined by expression of CD14, a lipopolysaccharide (LPS) receptor routinely used as a lineage marker for monocytes. CD14^+^ monocytes are the parent population for all monocyte populations/subsets described in this manuscript. Representative dot plots demonstrate gating strategies used in identifying the CD14^+^ parent population and specific monocyte subsets under the different treatment conditions (Figure 1).

### Statistical analyses

The frequencies of CD163^+^, M-CSF^+^, and IL-34^+^ populations in brain and the percent frequencies of M-CSF^+^ and IL-34^+^ monocyte (CD14^+^) populations from the *in vitro* studies were compared using ordinary and repeated measures one-way ANOVA with Tukey’s multiple comparisons post-test. Pearson correlations were performed to evaluate potential relationships between monocyte subsets investigated in the *in vitro* studies, as well as the results from the bioquantification of antigens of interest identified by immunohistochemistry in brain tissues. Statistical analyses were performed using Prism 6 for Mac OS X (GraphPad Software, version 6.0d). *P* values ≤0.05 were considered statistically significant.

## Results

### The frequency of CD163^+^ brain MΦs/microglia is increased in SIVE

**Figure 2:**
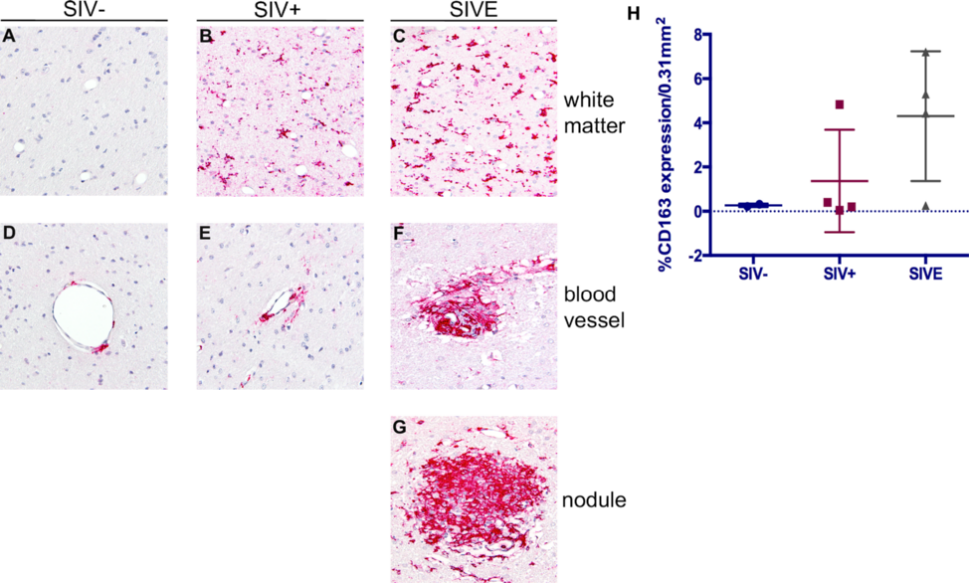
**CD163 is upregulated in brain of SIV-infected rhesus macaques with and without encephalitis (SIVE).** In the frontal white matter (FWM) of seronegative animals, CD163 detection (red) is limited to perivascular MΦs **(D)**. CD163^+^ perivascular MΦs are more frequently observed in SIV-infected animals **(E)**, with substantial perivascular cuffing of CD163^+^ MΦs in those with encephalitis **(F)**. In contrast to seronegative animals, few CD163^+^ cells are seen within the white matter parenchyma of animals with SIV infection but without encephalitis **(A)**. In rare SIV cases, infrequent areas in white matter show CD163^+^ microglia, many appearing to be in an activated state with thickened, retracted processes **(B)**. In SIVE, the number of parenchymal CD163^+^ cells greatly increases and appear to be highly activated **(C)**. Prominent accumulation of CD163^+^ cells is also observed within nodular lesions in SIVE **(G)**. All panels are shown under oil immersion at × 400 magnification. Bioquantification of parenchymal CD163^+^ MΦs/microglia reveals an increase in these cells in FWM of the majority of the animals that developed SIVE, as compared to seronegative and SIV infected animals without encephalitis **(H)**. Data points represent the average percent of parenchymal CD163 expression per animal of immunohistochemical staining in twelve randomly selected 0.31 mm^2^ fields of brain tissue from two seronegative (•), four SIV^+^ (■), and four SIVE (▲) animals. Perivascular cuffs and nodular lesions were excluded from quantification.

As others and we have shown previously, considerable accumulation of CD163^+^ MΦs and microglia is seen in SIVE brain [35,38-40] (Figure 2). In the brain of non-infected animals, CD163 expression is largely limited to the perivascular MΦ, with little to no immunopositivity detected in the parenchymal white matter (Figure 2A, D). Infrequent areas of CD163 expression are seen in the parenchymal white matter in SIV infection (Figure 2B); however, this appears to be considerably less than that observed in SIVE (Figure 2C). Likewise, a slight accumulation of CD163^+^ perivascular MΦs is seen in SIV-infected animals without encephalitis (Figure 2E), as compared to non-infected animals (Figure 2D), but without the significant cuffing characteristic of SIVE (Figure 2F). In addition to CD163 expression by MΦs accumulating perivascularly in SIVE brain, MΦs comprising nodular lesions are also CD163^+^ (Figure 2G).

Although microglial expression of CD163 is seen in the parenchymal white matter in SIV infection without encephalitis, it does appear to be greater in SIVE. To determine the extent of parenchymal CD163 expression in SIV and SIVE, we utilized a bioquantification software system to quantify parenchymal CD163 positivity in cerebral white matter of non-infected and SIV-infected rhesus macaques, with and without encephalitis, excluding perivascular cuffs and nodular lesions in SIVE. This reveals an overall marked accumulation of CD163^+^ MΦs and microglia in SIVE, as compared to the SIV without encephalitis and non-infected groupings (Figure 2H). It does not reach statistical significance, however, presumably due to the small number of animals in the study and the presence of a single outlier in each of the SIV^+^ and SIVE groupings.

### M-CSF, but not IL-34, expression is associated with CD163^+^ MΦs that accumulate perivascularly and within nodular lesions in SIVE

**Figure 3:**
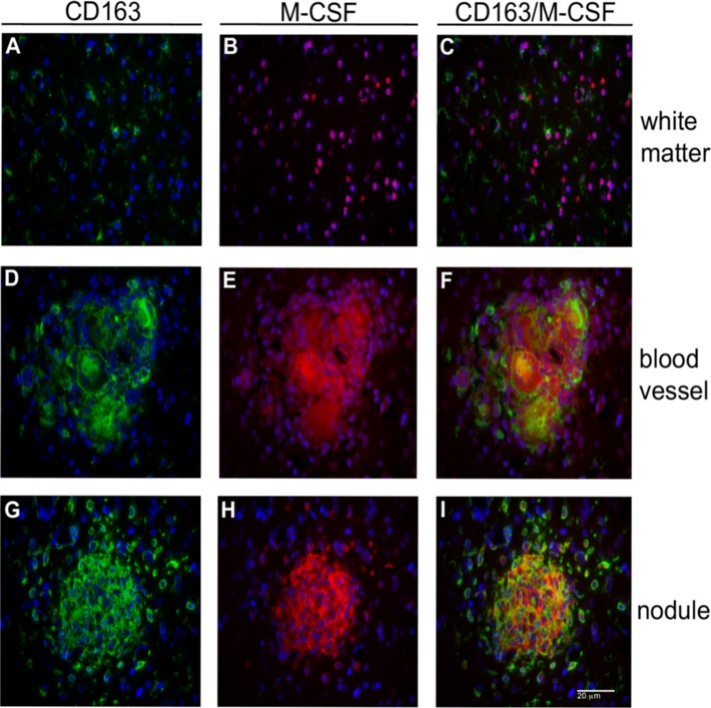
**Accumulating perivascular and nodular CD163** ^**+**^ **MΦs/microglia are a major source of M-CSF in SIVE.** Parenchymal CD163^+^ microglia **(A)** do not appear to express M-CSF in white matter **(C)**; however, M-CSF is produced by other glia **(B)**. Significant co-localization of CD163 **(D, G)** and M-CSF **(E, H)** is seen within perivascular cuffs and nodular lesions **(F, I)** and appears to be the principal source of M-CSF in SIVE brain.

Recently, we reported that both known ligands for cFMS, M-CSF and IL-34, are expressed in white matter of non-infected and SIV-infected rhesus macaques [27]. These studies showed that while IL-34 expression remained unchanged in cerebral white matter, irrespective of SIV infection or the development of SIVE, M-CSF expression appeared to be decreased by parenchymal cells in SIV and SIVE, but with significant expression by cells accumulating perivascularly and within nodular lesions in SIVE brain [27]. IL-34 also appeared to be expressed by some cells in these areas of pathology but to a markedly lesser degree than M-CSF [27]. To further characterize the IL-34 and M-CSF expressing cells in SIVE, we explored a potential relationship between M-CSF and/or IL-34 expression with CD163^+^ MΦs/microglia through double-label immunohistochemistry studies. These studies reveal little co-localization of CD163 (Figure 3A) and M-CSF (Figure 3B) in the parenchymal white matter (Figure 3C); however, M-CSF does appear to be produced by other CD163^−^ cells (Figure 3C). In contrast, significant co-localization of CD163 with M-CSF is seen in cells that accumulate perivascularly and within nodular lesions (Figure 3F,I).

**Figure 4:**
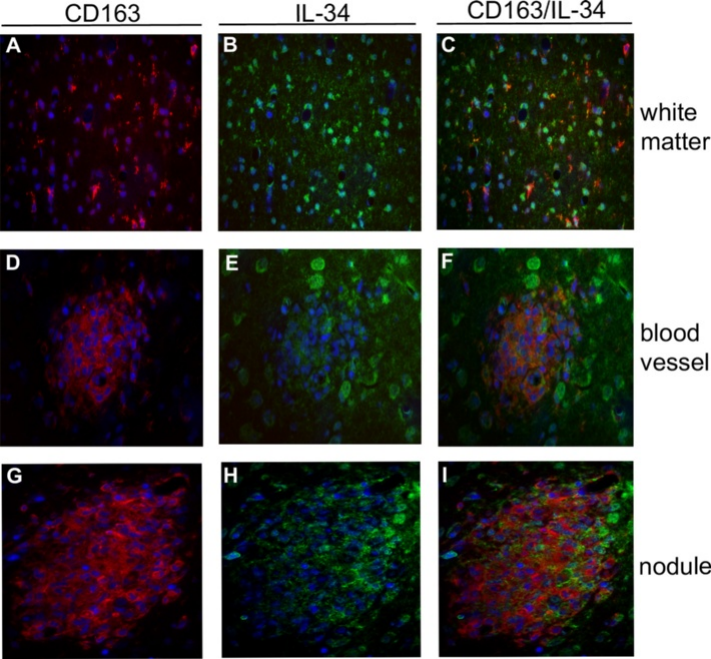
**Parenchymal CD163** ^**+**^ **microglia, but not perivascular or nodular CD163** ^**+**^ **MΦs, express IL-34 in FWM of rhesus macaques with SIVE.** In white matter, CD163^+^ microglia **(A)**, as well as other glia **(B)**, express IL-34 **(C)**. IL-34 expression appears to be limited to cells within the vicinity of perivascular cuffs **(E)**, as CD163^+^ MΦs that accumulate perivascularly **(D)** do not appear to express appreciable levels of IL-34 **(F)**. In contrast to perivascular cuffs, IL-34 is more highly expressed within nodular lesions **(H)**. These lesions are comprised largely of CD163^+^ MΦs **(G)**, however, little overlap with IL-34 is observed **(I)**.

In contrast to M-CSF, some CD163^+^ parenchymal microglia appear to express IL-34 in SIVE (Figure 4A,B,C). Additionally, numerous glia, not positive for CD163, also appear to express IL-34 (Figure 4C). While IL-34 expression is seen in MΦs/microglia in the vicinity of perivascular cuffs (Figure 4E), it is not seen to a great extent by cells that comprise the cuffs, themselves (Figure 4F). As compared to cuffs, IL-34 is more highly expressed within nodular lesions (Figure 4H); however, little overlap with CD163 is seen (Figure 4I).

### M-CSF and IL-34 are normally expressed by neurons in brain, which is altered with SIV infection

**Figure 5:**
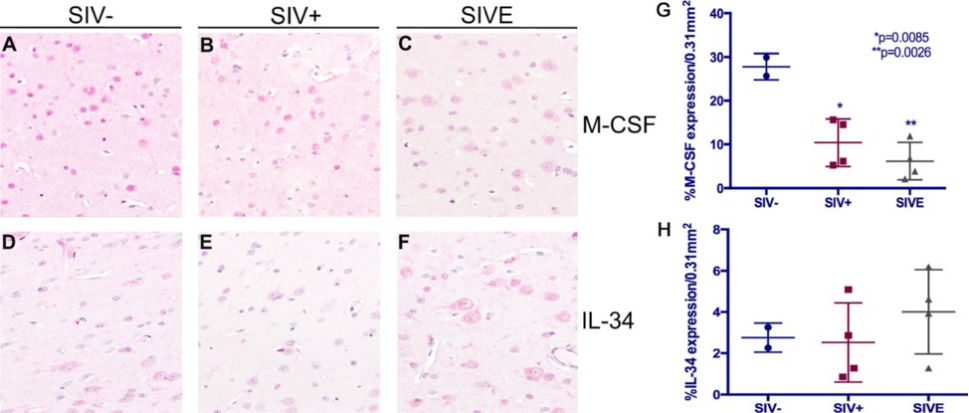
**Normal neuronal M-CSF and IL-34 expression is altered in SIV and SIVE.** Cells with neuronal morphology in frontal grey matter (FGM) of rhesus macaques express M-CSF (**A**, **B**, **C**; red) and IL-34 (**D**, **E**, **F**; red), regardless of the presence of SIV infection. Panels are shown at ×400 under oil*.* Bioquantification analyses reveal neuronal M-CSF expression is significantly decreased in brain of SIV-infected animals, with (*P* = 0.0026) and without (*P* = 0.0085) encephalitis, as compared to seronegative controls **(G)**. In contrast to M-CSF, IL-34 quantification suggests an increase in neuronal IL-34 expression in animals with SIVE, as compared to seronegative and SIV-infected animals **(H)**; however, this did not reach statistical significance in this small study. Data points represent the average percent expression per animal of immunohistochemical staining in twelve randomly selected 0.31 mm^2^ fields of brain tissue from two seronegative (•), four SIV^+^ (■), and four SIVE (▲) animals.

In addition to M-CSF and IL-34 expression by cells in cerebral white matter, neurons also express both cytokines in brain of non-infected (Figure 5A,D), as well as SIV-infected rhesus macaques without and with encephalitis (Figure 5B,C,E,F). Bioquantification of neuronal M-CSF expression revealed that it is significantly decreased in SIV infection, with an even greater reduction seen in those with encephalitis (Figure 5G). In contrast, bioquantification of IL-34 expression by neurons shows an overall increase in expression by animals with SIVE (Figure 5H), as compared to those without encephalitis and non-infected animals; however, this does not reach statistical significance in this small study.

### cFMS signaling alters CD16^+^CD163^+^ monocyte frequency *in vitro*

**Figure 6:**
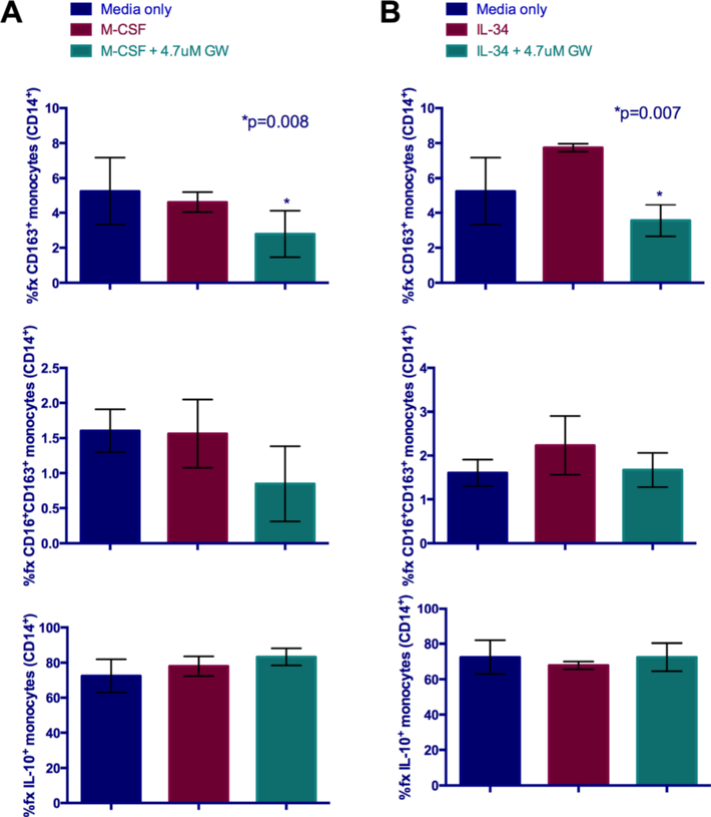
**cFMS inhibition alters the frequency of CD16** ^**+**^ **CD163** ^**+**^ **rhesus macaque monocytes** ***in vitro*** **.** An increase in the frequency of CD163^+^ and CD16^+^CD163^+^ was observed with IL-34 **(B)** but not M-CSF treatment **(A)**, as compared to PBMC cultured in the presence of media only. Interestingly, the frequencies of these monocyte subsets were reduced by the addition of the tyrosine kinase inhibitor (TKI), GW2580, which may reflect inhibition of endogenous M-CSF and/or IL-34 activity in the culture. The frequency of IL-10^+^ monocytes was high, regardless of treatment.

Previous studies have demonstrated that M-CSF promotes expression of CD16 by monocytes *in vitro* [41,42], which as we have shown co-localizes markedly with CD163 by perivascular MΦs and parenchymal microglia in the brain of patients with HIVE [35]. To begin to understand the mechanisms driving CD16^+^CD163^+^ expression by MΦs and microglia in HIVE and SIVE, we examined the effect of M-CSF and IL-34, which also exerts its biological effects through the same M-CSF receptor, cFMS receptor tyrosine kinase, on rhesus macaque and human monocyte subset frequencies *in vitro*. PBMC, isolated from whole blood of two non-infected, healthy rhesus macaques and one human buffy coat, were cultured in complete RPMI containing 2% FBS and supplemented with 2.5 ng/ml M-CSF or IL-34 with and without GW2580, a tyrosine kinase inhibitor (TKI) reported to have high specificity for their shared receptor, cFMS. After 24 h, harvested cells were analyzed by flow cytometry for alterations in CD16 and CD163 expressing monocyte subsets (Figure 1). These studies demonstrated no effect of M-CSF exposure on CD163^+^ or CD16^+^CD163^+^ rhesus macaque monocyte (CD14^+^) frequency, as compared to those without M-CSF treatment (Figure 6A). Interestingly, PBMC cultured in the presence of M-CSF and the cFMS inhibitor, GW2580, did show a decrease in the frequency of both monocyte subsets that reached statistical significance for the CD163^+^ population (Figure 6A). Although it is unclear what may have influenced the monocyte subset frequencies in non-treated cultures, the finding that inhibiting cFMS signaling reduces these frequencies supports a role for cFMS in their expansion. No influence on IL-10 expression was observed with M-CSF exposure or cFMS inhibition (Figure 6A).

In contrast to M-CSF, IL-34 treatment of rhesus macaque PBMC did increase both the CD163^+^ and CD16^+^CD163^+^ monocyte subset frequencies, but these increases did not reach statistical significance (Figure 6B). Like M-CSF, the IL-10^+^ monocyte frequency remained unchanged with IL-34 exposure (Figure 6B).

**Figure 7:**
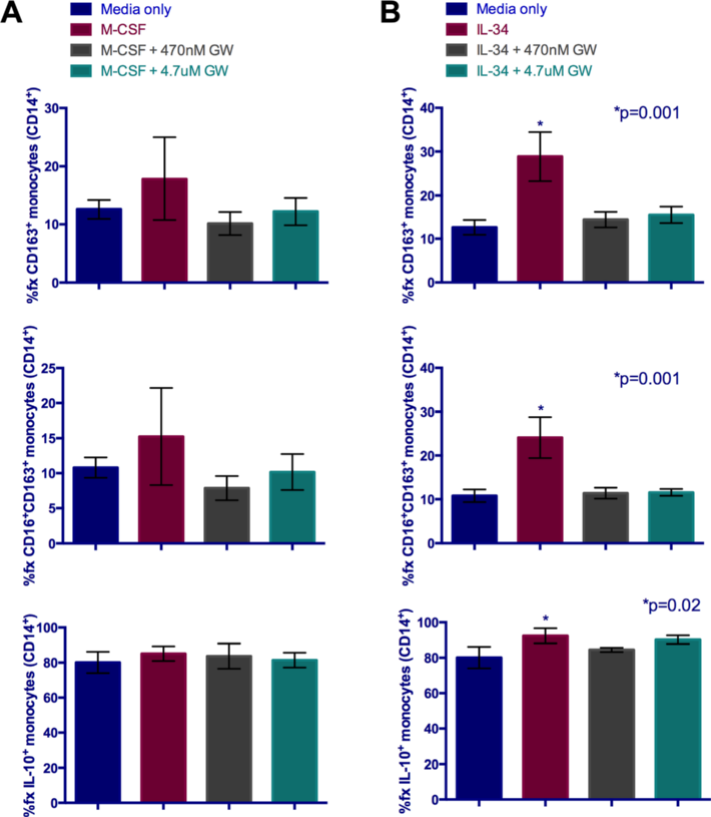
**M-CSF and IL-34 increase the frequency of CD16** ^**+**^ **CD163** ^**+**^ **human monocytes** ***in vitro*** **, which is reversed by inhibition of cFMS signaling.** An increase in the frequency of CD163^+^ and CD16^+^CD163^+^ monocytes is observed with both M-CSF **(A)** and IL-34 **(B)** but only reaches statistical significance with IL-34 treatment **(B)**. This may reflect the reportedly high affinity IL-34 has for cFMS, suggested by decreased standard deviation seen among replicates treated with IL-34, as compared to those treated with M-CSF. The increased frequencies of these monocyte subsets are abrogated by the addition of GW2580, which restores the frequencies to percentages at or near those seen in PBMC cultured in media only. The frequency of IL-10^+^ monocytes is high, regardless of treatment; however, a small but statistically significant increase is seen with IL-34.

*In vitro* studies utilizing human PBMC showed that both cytokines increased the frequency of CD163^+^ and CD16^+^CD163^+^ monocytes (CD14^+^), as compared to cells cultured without cytokine supplementation (Figure 7). Due to a greater number of PBMC recovered from the human buffy coat than the rhesus macaques, we investigated two different concentrations of GW2580 for its ability to counter these phenotypic alterations exerted on monocytes by M-CSF and IL-34. Both concentrations were able to effectively reduce the frequency of CD163^+^ and CD16^+^CD163^+^ monocytes cultured in the presence of either cytokine (Figure 7A,B), strongly supporting the notion that cFMS signaling promotes CD163 and CD16 expression. Although CD16 and CD163 expression by M-CSF treated monocytes is markedly changed with treatment or cFMS inhibition, statistical significance is not reached due to variability seen among cultures, which were performed in triplicate. In contrast, statistical significance is seen with IL-34 treatment and likely reflects the higher affinity IL-34 has for cFMS [43]. Although, like the rhesus macaque PBMC studies, no difference in IL-10^+^ monocyte subset frequency was seen with M-CSF treatment, a small, but statistically significant increase in this population was observed with IL-34 treatment (Figure 7B).

### The frequency of IL-34 expanded CD163^+^ monocytes correlates with IL-10^+^ monocytes *in vitro*

**Figure 8:**
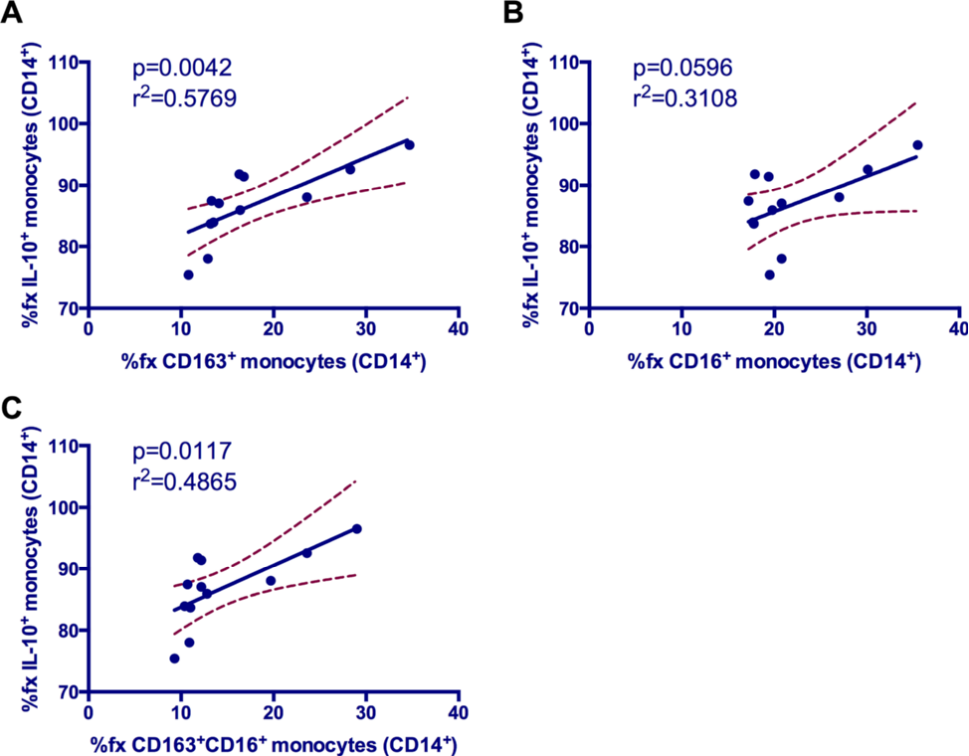
**The frequency of IL-10** ^**+**^ **monocytes correlates with increased frequencies of specific monocyte subsets when treated** ***in vitro*** **with IL-34.** Increased frequencies of CD163^+^ **(A)** and CD16^+^CD163^+^ **(C)** monocyte subsets were found to correlate significantly with IL-10^+^ monocyte frequencies. The most significant relationship with IL-10^+^ monocytes was seen with the frequency of CD163^+^ monocytes, with approximately 58% of the data points falling within the correlation. This is in agreement with the notion that CD163 expression phenotypically defines M2 activation of monocytes/MΦs. The frequencies of CD16^+^ **(B)** monocytes do not correlate with IL-10^+^ monocyte frequencies.

Because a small but statistically significant increase in the IL-10^+^ monocyte subset frequency was observed with IL-34 treatment, additional statistical analyses were employed to investigate potential relationships between IL-10^+^ monocyte subset frequencies and the frequencies of other monocyte subsets of interest. Pearson’s correlations revealed a fairly strong correlation between the frequencies of CD163^+^ and IL-10^+^ monocytes (Figure 8A). This relationship remains significant when further fractionated into CD163^+^ monocytes that also express CD16 (Figure 8C), but not when compared to CD16^+^ monocyte frequencies not subfractionated by CD163 expression (Figure 8B).

## Discussion

In earlier work, we found that the CD163^+^ MΦs that accumulate perivascularly and within nodular lesions also appear to be the principal reservoir of productive HIV infection in the brain [28]. More recently, we reported that the principal source of M-CSF in SIVE are the MΦs that accumulate perivascularly and within nodular lesions [27]. Here, we demonstrate that these MΦs are also CD163^+^. We previously suggested that the MΦs/microglia that accumulate perivascularly and within nodular lesions in HIV have recently entered the CNS from the peripheral blood [28,44]. As such, peripheral blood monocytes/MΦs may be the major source of M-CSF in the brain in HIV infection, which is elevated in CSF of individuals with HIV infection and impaired cognition [24,25].

The role of M-CSF in HIV infection and associated CNS disease is unclear. Here, we demonstrate that M-CSF and IL-34, which is also expressed in brain and shares the same receptor, cFMS, promotes expansion of the CD16^+^CD163^+^ monocyte subset. Inhibiting cFMS signaling with a small molecule TKI with high specificity for cFMS attenuates this expansion. These CD16^+^CD163^+^ monocytes are phenotypically similar to monocytes we find expanded in circulation of viremic HIV-infected persons [34]. Moreover, they are similar to MΦs/microglia that accumulate in the brain of patients with HIV infection with and without encephalitis [18,28,34,35]. Although co-localization of CD16 and CD163 is observed on both parenchymal microglia and MΦs comprising perivascular cuffs and nodular lesions, it is the perivascular and nodular MΦs that appear to be the primary reservoir of productive virus in the brain [28,35]. Interestingly, MΦs accumulating in these regions are also the principal source of M-CSF in SIVE brain. *In vitro* studies have demonstrated M-CSF enhances the susceptibility of MΦs to HIV infection and promotes virus replication [45-47]. In turn, HIV infection of MΦs promotes production of M-CSF [45], constituting a positive feedback loop that supports HIV production/infection. In addition to its natural role as a key factor in MΦ survival, M-CSF may serve as a key factor in the development of MΦs as a long-lived cellular reservoir of HIV/SIV infection. Together, these findings support a pathological role for M-CSF in both the development and establishment of tissue reservoirs of HIV-infected MΦs.

M-CSF may also contribute to the development and maintenance of tissue reservoirs, as well as neuropathogenesis, by enhancing migration of monocytes/MΦs, some of which may be infected, into the brain. In support of this hypothesis, we previously reported that the MΦs comprising perivascular cuffs and nodular lesions also express markers that suggest recent entry from the peripheral blood into the CNS compartment [28,34]. M-CSF has been shown to stimulate monocyte migration *in vitro* and *in vivo*, which is mediated through the association of PI3K with phosphorylated Y721 of the activated receptor, cFMS [48,49]. Enhanced CD163 expression may also contribute to the invasiveness of these cells, as CD163 has been shown to augment monocyte adherence to LPS or cytokine-stimulated endothelial cells [50].

M-CSF expression is also seen in CD163^−^ cells in the parenchymal white matter, although this appears to be less than that expressed by cells within perivascular cuffs and nodular lesions. These cells have not yet been identified, but may be identified as microglia that do not express CD163 or express it below the level of detection in this study. Alternatively, these may be astrocytes, as murine astrocytes have been demonstrated to express M-CSF *in vitro* when stimulated with IL-1 or TNFα [51], cytokines elevated in CSF and brain in HIV infection and believed to contribute to HIV-associated neurological impairment [52-54].

In contrast to M-CSF, IL-34 glial expression appears to be largely limited to parenchymal microglia, rather than perivascular and nodular MΦs in SIVE. Additionally, IL-34 expression is largely unchanged from that observed in brain of non-infected animals and those with SIV infection but without encephalitis [27], suggesting a spatial relationship between the two cytokines in brain and, presumably, different biological roles. In support of this notion, we find that both M-CSF and IL-34 are normally expressed by neurons, however, while neuronal M-CSF expression is decreased in SIVE, IL-34 may be increased.

Although M-CSF and IL-34 appear to have some shared roles, differences have been reported in signaling kinetics and function [43] and may exert different biological effects in response to foreign pathogens in the brain and/or maintaining brain homeostasis. IL-34 is highly expressed in brain and is a key regulator in neuronal progenitor and microglial maturation in the developing brain [55,56] and may contribute to the survival of these cells. Systemic administration of M-CSF or IL-34 in a mouse model of kainic acid-induced neurotoxicity showed a reduction in neuronal loss that appears to be a direct result of neuronal cFMS signaling [57]. In addition, activation of astrocytes and microglia also appears to be attenuated in response to M-CSF or IL-34 administration in this model [57]. This may be the result of reduced neurotoxicity, itself, affecting gliosis and/or may be a direct affect of these cytokines on astrocytes and/or microglia. In support of this notion, IL-34 treatment of microglia *in vitro* promotes expression of the anti-inflammatory factor, tumor growth factor-β (TGF-β) [58]. It is feasible that both mechanisms contribute to neuroprotection. As such, upregulation of IL-34 by injured or stressed neurons in SIVE may be a mechanism by which neurons promote self-protection through autocrine IL-34 signaling, as well as by communicating to neighboring microglia to reduce inflammatory factors that would promote activation of surrounding glia and advance damage to neurons. Interestingly, our *in vitro* studies of human PBMC showed a small but statistically significant increase in IL-10 expression by monocytes. Although additional studies are needed to assess the potential biological relevance of this finding, the frequency of CD163^+^ monocytes correlates strongly with the frequency of monocytes positive for IL-10. If these cells are also a rich source of IL-10 in the brain, their continued presence throughout the disease process may suggest a mechanism for neuropathogenesis that involves an immunosuppressive environment in the CNS that is tolerant of HIV/SIV and opportunistic pathogens, while contributing to neuropathogenesis through chronic inflammatory activation. The significant variability of neuronal IL-34 expression in SIV-infected animals without encephalitis may reflect other pro- or anti-inflammatory factors not investigated in this study.

## Conclusions

Tissue MΦs are increasingly recognized as an important reservoir of viral persistence in HIV infection. In the brain, infected MΦs and microglia pose additional concerns due to the presence of virus and viral proteins, as well as chronic inflammation in the CNS, all of which contribute to the development and progression of HIV-associated neurocognitive impairment. Here, we demonstrate substantial M-CSF expression by CD163^+^ MΦs that accumulate perivascularly and within nodular lesions in SIVE, suggesting a role for M-CSF in the development and maintenance of long-lived viral MΦ/microglial reservoirs in the brain, as well as supporting M2 activation of perivascular MΦs and microglia. In contrast to M-CSF expression in brain, glial IL-34 expression appears to be unchanged between healthy and infected animals; however, neuronal IL-34 expression is actually increased in those with SIVE. As such, both cytokines may contribute to this MΦ/microglial phenotype, which is consistent with M2 MΦ activation.

**Figure 9:**
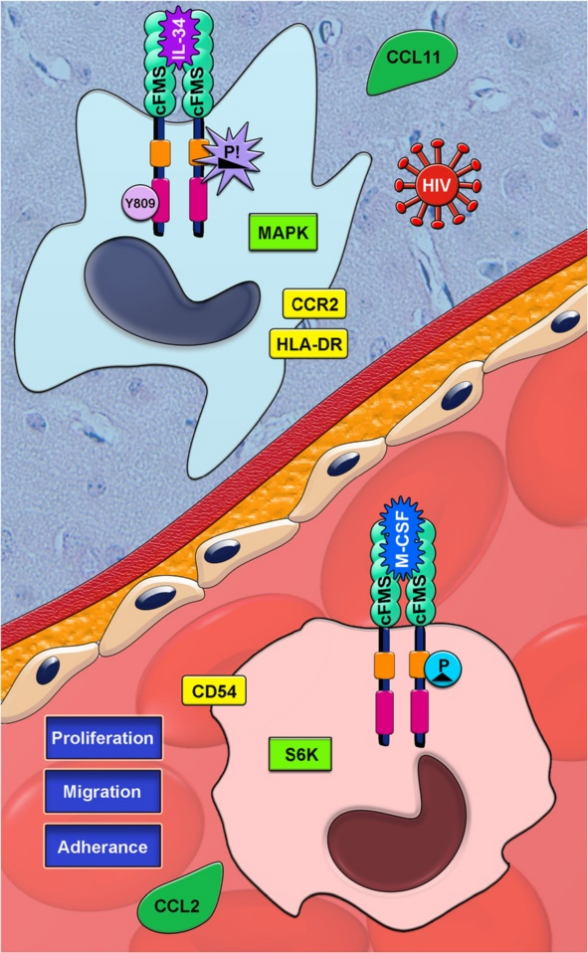
**M-CSF and IL-34 reportedly exert different biological effects on monocyte/MΦs.** The limited studies comparing M-CSF and IL-34 in parallel report differences in signaling, function, and location of expression. IL-34 appears to be the dominant cFMS ligand in tissues (left, blue MΦ) [56,59], where expression of M-CSF is associated more strongly with the peripheral blood and bone marrow (right, red monocyte) [62]. Upon ligation of cFMS, both cytokines phosphorylate the same intracellular tyrosine residues [43,59]; however, IL-34-mediated phosphorylation (wedge) may occur more rapidly than that mediated by M-CSF (isosceles triangle) [43]. Further, IL-34 signaling appears to decay more quickly [43] but stimulates more receptors, as compared to M-CSF [43], with the exception of Y809, which may be favored by IL-34 [43]. Following activation of second messengers, IL-34-induced signal transduction has been reported to more strongly activate the mitogen-activated protein kinases but has a weaker effect on the ribosomal S6 kinases than M-CSF [43]. Both ligands participate in chemokine-mediated chemotaxis of monocytes/MΦs through upregulation of different mediators. IL-34 upregulates C-C chemokine receptor type 2 (CCR2) by primary human monocytes [63], and M-CSF promotes expression of its ligand, chemokine (C-C motif) ligand 2 (CCL2) [43]. Additionally, IL-34, but not M-CSF, promotes production of CCL11 [43], while the adhesion molecule, CD54, is upregulated by primary human monocytes more strongly by M-CSF [63]. Functionally, M-CSF, more than IL-34, promotes migration and supports proliferation in murine myeloid cell lines [43]. IL-34, however, promotes greater expression of HLA-DR [43] and may play a greater role in promoting cell-mediated immunity. In the context of productive HIV infection, both IL-34 and M-CSF promote virus production but are greater in the presence of IL-34 [43]. Together, these limited findings suggest that both cFMS ligands promote distinct populations of myeloid cells that potentially have shared, as well as specialized functions specific to M-CSF or IL-34 stimulation.

The role of neuronal IL-34 is not clear; however, it appears to be an important factor in the developing brain [59], possibly through supporting neuronal survival. In recent work, Luo *et al*. demonstrated that systemic administration of either cFMS ligand is neuroprotective in a transgenic mouse model of Alzheimer’s disease [57]. As neurons, astrocytes, oligodendrocytes, and microglia may all express the receptor, cFMS [57,60,61], it is unclear if this is a direct and/or indirect effect of M-CSF and/or IL-34 on neurons. Here, we find that while neuronal IL-34 is significantly increased in SIVE as compared to non-infected animals and those with SIV but without encephalitis, neuronal M-CSF expression progressively decreases in SIV and SIVE. Together, these findings of IL-34 and M-CSF expression in the brain support the idea of at least some distinct functions between the two cytokines, as well as a spatial relationship that is complementary, rather than competitive (Figure 9).

While additional studies are needed to elucidate the potentially divergent roles of M-CSF and IL-34 in HIV-associated neurodegenerative disease, these studies suggest that cFMS signaling through ligation of M-CSF plays an important role in its pathogenesis. IL-34 may also contribute to M2 activation/polarization in the brain but may also promote neuronal survival. Advancing our current understanding of the role of these partially redundant cytokines in brain health and disease will aid in the development of targeted therapeutic strategies aimed at eliminating MΦ reservoirs of HIV infection in the brain and other tissues.

## Abbreviations

CNS: Central nervous system
CSF: Cerebrospinal fluid
FGM: Frontal grey matter
FWM: Frontal white matter
HAND: HIV-associated neurocognitive disorders
HIV: Human immunodeficiency virus
HIV-D: HIV-associated dementia
HIVE: HIV encephalitis
IL: Interleukin
M1: Classically activated MΦs
M2: Alternative/type 2 activated MΦs
M-CSF: Macrophage-colony stimulating factor
MΦ: Macrophage
PBMC: Peripheral blood mononuclear cells
ROS: Reactive oxygen species
SIV: Simian immunodeficiency virus
SIVE: SIV encephalitis
T_H_: T helper
TKI: Tyrosine kinase inhibitor
